# Silk fibroin/hydroxyapatite composite hydrogel induced by gamma-ray irradiation for bone tissue engineering

**DOI:** 10.1186/s40824-017-0098-2

**Published:** 2017-06-24

**Authors:** Min Hee Kim, Beom Su Kim, Jun Lee, Donghwan Cho, Oh Hyeong Kwon, Won Ho Park

**Affiliations:** 1 Department of Advanced Organic Materials and Textile Engineering System, College of Engineering, Chungnam National University, Daejeon, 34134 South Korea; 2 Wonkwang Bone Regeneration Institute, Wonkwang University, Iksan, South Korea; 3 Bone Cell Biotech, Daejeon, South Korea; 4 Department of Polymer Science and Engineering, Kumoh National Institute of Technology, Gumi, South Korea

**Keywords:** Silk fibroin, Hydroxyapatite, Irradiation, Hydrogel, Bone tissue engineering

## Abstract

**Background:**

In this study, silk fibroin (SF) composite hydrogels containing hydroxyapatite (HAP) nanoparticles (NPs) for bone tissue engineering were fabricated using gamma-ray (γ-ray) irradiation treatment. During the irradiation, the HAP dispersed SF solution was changed to the chemically crosslinked SF hydrogel.

**Methods:**

Distribution of HAP NPs in the SF hydrogel was examined by SEM imagery and energy dispersive X-ray spectrophotometry, and the crystalline structure of SF composite hydrogels was also confirmed by X-ray diffractometry. An optimum preparation condition of the SF/HAP composite hydrogels was determined with various HAP contents. For evaluation of the osteogenic differentiation of human mesenchymal stem cells (hMSCs), alkaline phosphatase activity (ALP), HAP nucleation in SBF and in vitro calcium accumulation were measured.

**Results:**

The results revealed that compared with the pure SF hydrogels, the SF/HAP composite hydrogels improved osteogenic differentiation.

**Conclusion:**

This paper demonstrates the great potential of the SF/HAP composite hydrogels in terms of the production of the bone tissue engineering scaffolds for which osteogenesis is required.

## Background

Bones provide mechanical protection for the body (such as protecting internal organs and blood forming marrow), facilitate locomotion, and serve as a reservoir for calcium, magnesium and phosphate minerals [1]. Osteogenesis often requires a replacement graft to restore the function of damaged tissue. Scaffolds for bone tissue engineering offer a promising alternative treatment for medical use, as well as a controllable system for studies of biological function, development of biology and pathogenesis [2, 3]. The materials for scaffolds exhibit many of the mechanical properties of the engineered graft. Inorganic and organic scaffolds are easily fabricated into different structures, but the compressive modulus of organic scaffolds is often unsatisfactory. Alternatively, ceramic scaffolds have excellent stiffness, but are fragile and have low porosity, resulting in loosening of fractured implants in clinical applications. Combining organic and inorganic materials to form composite scaffolds can enhance the mechanical and biochemical properties of scaffolds for bone tissue regeneration [4–6].

Numerous research efforts have addressed the development of an ideal scaffold for bone tissue engineering [7, 8]; however, they still have several limitations. Due to its biocompatibility, biodegradability, controllable strength, and good oxygen and water permeability, silk fibroin (SF) originated from *Bombyx mori* has been fabricated for various tissue engineering scaffolds with various chemical, structural and biochemical modifications. SF has been investigated with regard to applications of tissue engineered blood vessels, skin, bone, and cartilage [9–13]. Porous 3-D scaffolds are suitable for bone tissue engineering, as they enhance cell viability, proliferation, and migration. Furthermore, highly porous scaffolds (up to 92% porosity) facilitate nutrient and waste transport into and out of the scaffolds [14]. Physically crosslinked SF hydrogels have been produced through the induction of the β-sheet structure in SF solutions. However, due to the β-sheet formation, the SF exhibits relatively slow degradation in vitro and in vivo. To improve the degradability and strength of hydrogels, the SF has been crosslinked in recent years via a number of methods. Chemically crosslinked SF hydrogels using chemical crosslinkers, such as genipin and glutaraldehyde [10, 15, 16], ionizing irradiation [17], nitrate salts [18], and enzymatic crosslinker including tyrosinase [19] have also been studied. However, these crosslinking methods were found to be time-consuming and cytotoxic. Therefore, it is very important to establish a rapid crosslinking method to develop chemically crosslinked SF hydrogels.

Ionizing radiation, like gamma ray (γ-ray), electron beam, and ion beam has been used as an initiator for the preparation of hydrogel from unsaturated compounds. The irradiation results in the formation of radicals on the unsaturated polymer chain and water molecules, which attack the polymer chains and thus induce intermolecular crosslinking [20, 21]. The ionizing radiation would be an excellent pathway for the preparation of uniformly dispersed organic/inorganic composite hydrogels, because polymer solutions easily undergo chemical crosslinking and solidify immediately. In addition, potentially toxic initiators and crosslinkers do not need to be used for the synthesis of organic/inorganic composite scaffolds for tissue engineering [22].

This study employed SF and HAP NPs due to the composite hydogel’s biocompatibility and osteoconductivity, and easy reproducibility of fabrication. The SF hydrogels were prepared via a chemical crosslinking reaction using γ-ray irradiation. Also, the effects of HAP content on the morphological, structural, and mechanical properties of porous SF hydrogels were examined. In addition, the effect of SF/HAP composite hydrogel on the osteogenic responses of hMSCs was assessed with respect to bone tissue regeneration.

## Methods

### Preparation of SF solution

SF solution was prepared according to the previously established protocol [17, 23]. Briefly, the scoured *Bombyx mori* (*B. mori)* SF fiber was dissolved in a ternary solvent composed of calcium chloride, ethanol and water (1:2:8 M ratio) at 85 °C for 4 h. The dissolved SF solution was dialyzed in distilled water for 72 h using dialysis cellulose tubular membranes (250-7 μ, Sigma, St. Louis, MO, USA) to remove the salts. After dialysis, the solution was centrifuged at 3000 rpm for 10 min to remove the insoluble impurities. The final concentration of the resultant aqueous SF solution was approximately 2.3 wt%, which was determined by weighing the remaining sponge weight after lyophilization. A higher concentration SF solution was prepared by reverse dialysis against 25 wt% polyethylene glycol (PEG, M_w_ 20,000) solution at room temperature [24, 25]. The SF concentration after reverse dialysis was approximately 7.9 wt%. The regenerated SF solution was stored at 4 °C for further use.

### Preparation of SF/HAP composite hydrogels

**Fig. 1:**
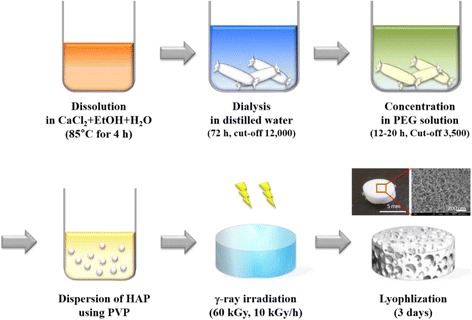
Schematic illustration of the preparation method of the SF/HAP composite hydrogels

SF/HAP composite hydrogels were prepared as shown in Fig. 1. Freshly regenerated 7.9 wt% SF solution was blended with poly(vinyl pyrrolidone) (PVP) to improve the dispersity of HAP NPs. SF/HAP aqueous solution was prepared by adding HAP NPs (particle size <200 nm, Sigma Aldrich, St. Louis, MO) with various concentration directly into the SF aqueous solution. SF/HAP aqueous solution was poured into a petri dish and irradiated by γ-ray from a Co-60 source. The irradiation dose varied to 60 kGy and the dose rate was 15 kGy/h. The irradiated samples were cut into small pieces and then lyophilized for 3 days to analyze various properties.

SF/HAP composite hydrogels with different HAP contents (0–3 wt%) were named as SF-0, SF-1, SF-2, and SF-3 respectively. Table 1 shows the compositions of SF/HAP composite hydrogels.

**Table 1:** Sample code and composition of SF/HAP composite hydrogels

Sample name	SF concentration (wt%)	HAP concentration (wt%)	PVP concentration (wt%)
SF only	7.9	0.0	1.0
SF-1% HAP	7.9	1.0	1.0
SF-2% HAP	7.9	2.0	1.0
SF-3% HAP	7.9	3.0	1.0

### Characterization

The pore structure, morphology, and distribution of HAP NPs of SF/HAP composite hydrogels were observed by field emission scanning electron microscopy (FE-SEM) (JSM-7000F, JEOL, Japan) and energy dispersive X-ray spectroscopy (EDX) equipment. The pore parameters including surface area, pore volume, pore size and porosity were characterized by mercury porosimetry (Micromeritics, ASAP 2020). The crystalline structure of SF/HAP composite hydrogels was measured by X-ray diffraction (XRD) (D8 Discover, Bruker, USA) in the range of 2θ from 5 to 50° (λ = 0.154 nm, 40 kV, 40 Ma). The compressive strength of composite hydrogels was measured using a cube-shaped sample (10 mm × 10 mm × 10 mm) by Instron 5848 mechanical tester machine with a crosshead speed of 5 mm/min and 50% strain using a 500 N load cell.

### Cell culture and proliferation assay

To evaluate the biocompatibility of composite hydrogel, hMSCs were purchased from the American Type Culture Collection (ATCC, Manassas, VA, USA). The cells were cultured in α-MEM (Gibco-BRL, Gaithersbug, MD, USA) containing 10% fetal bovine serum (FBS) and 1% antibiotics at 37 °C under 5% CO_2_ and 100% humidity. Osteoblast differentiation was induced using osteoblast differentiation reagents (10 mM *β*-glycerophosphate, 50 μg/mL ascorbic acid, and 100 nM dexamethasone (Sigma-Aldrich, St. Louis, MO, USA). The number of viable cells was determined using the CellTiter96^®^ aqueous one solution kit (Promega, Madison, WI, USA). Briefly, cells were seeded to the hydrogel. At a predetermined time point (6 days), 200 μL of MTS reagent was mixed with 500 μL of culture media and added to each well. After incubation for 2 h, absorbance of the supernatant was measured at 490 nm using an ELISA reader (SpectraMAX M3; Molecular Devices, Sunnyvale, CA, USA). After 6 days of cultivation, cell-loaded hydrogels were rinsed with PBS to remove the phenol red, and were with PBS. In addition, the Live/Dead^®^ Viability/Cytotoxicity staining kit (Molecular Probe, Eugene, OR, USA) reagent solution was added. After incubation for 30 min in a CO_2_ incubator, the samples were observed using an inverted fluorescence microscope (DM IL LED Fluo; Leica Microsystems, Wetzlar, Germany). SEM was used to observe cell adhesion to the hydrogels. After 6 days of culture, the cell-loaded hydrogels were fixed with 2.5% glutaraldehyde, and additional-fixation was performed with 0.1% osmium tetroxide (Sigma-Aldrich, St. Louis, MO, USA). After dehydration with a graded ethanol series (50%, 75%, 95% and 100%), the samples were sputter-coated with gold, and observed by SEM (EM-30; Coxem, Daejeon, Korea) [26].

### Alkaline phosphatase activity assay and in vitro hydroxyapatite nucleation

The degree of osteoblast differentiation in the cells was evaluated by determining the alkaline phosphatase (ALP) activity. After 7 days of culture using osteogenic induction medium, the adherent cells were removed from the hydrogel by homogenization in PBS with 1% Triton X-100. Then, the suspension was mixed with 0.1 M glycine NaOH buffer (pH 10.4) and 15 mM *p*-nitrophenyl phosphate (*p*-NPP; Sigma, St. Louis, MO, USA). After 30 min incubation at 37 °C, the reaction was terminated by adding 0.1 N NaOH, and the *p*-NPP hydrolysis was determined by ELISA reader (Spectra MAX M3) at 410 nm. Protein concentrations were measured by bicinchoninic acid (BCA) protein assay reagent kit (Pierce, Rockford, IL, USA), and normalized. To determine the hydroxyapatite nucleation on the surface of hydrogel, simulated body fluid (SBF) was used. Briefly, the fabricated hydrogels were immersed in 1× SBF (Biosesang, Sungnam, Korea), and maintained at 37 °C. After immersion period of 7 days, the hydrogels were removed from the fluid, gently rinsed with distilled water, and dehydrated with a graded ethanol series. After the sample was sputter-coated with gold, the behavior of hydroxyapatite crystal growth was observed by SEM (EM-30).

### In vitro calcium accumulation

hMSCs were cultured with continuous treatment with osteoblast differentiation reagents contained media. After 21 days, the cell-loaded hydrogels were fixed with 70% ice-cold ethanol for 1 h at 4 °C. After the ethanol was removed, calcium accumulation was measured by staining with 40 mM Alizarin Red-sulfate (AR-S; Sigma-Aldrich, St. Louis, MO, USA) solution, and normalized with non-cultured scaffold, respectively. The stained portions were photographed by digital camera. The deposited stain was then dissolved using 10% cetylpyridinium chloride solution and the absorbance was read at 562 nm by ELISA reader.

## Results and discussion

### Morphology and crystalline structure of the SF/HAP composite hydrogels

**Fig. 2:**
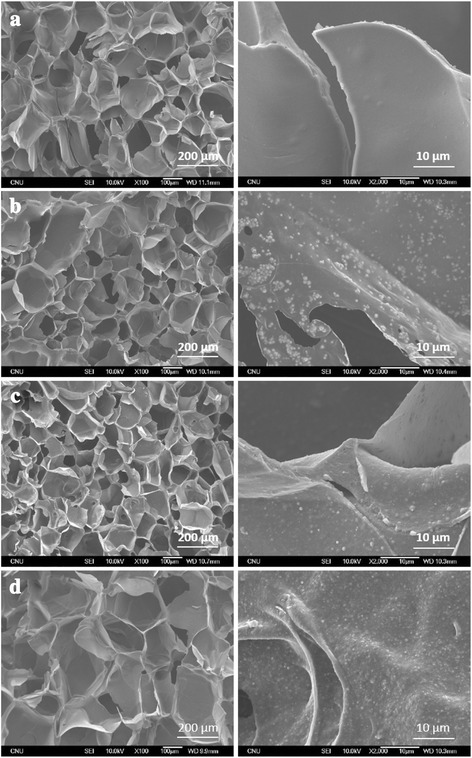
Representative FE-SEM images of **a** SF only, **b** SF-1% HAP, **c** SF-2% HAP, and **d** SF-3% HAP

**Fig. 3:**
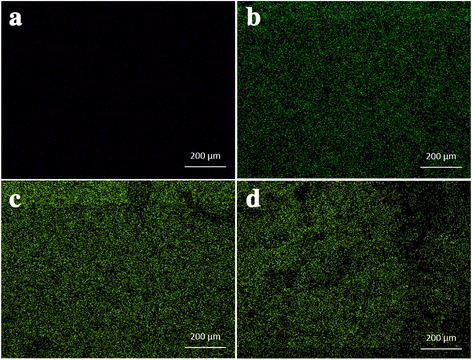
Distribution of calcium element in SF/HAP composite scaffolds; **a** SF only, **b** SF-1% HAP, **c** SF-2% HAP, and **d** SF-3% HAP

**Fig. 4:**
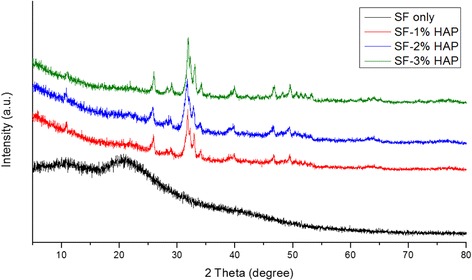
X-ray diffraction of SF/HAP composite scaffolds

The fabrication of 3-dimensional porous SF/HAP composite hydrogels was prepared by γ-ray irradiation process. The pore structure of each hydrogel was observed by FE-SEM (Fig. 2). Each hydrogel had uniform pore size and interconnected pore structure, in particular, HAP concentration did not affect the pore size within the hydrogels. HAP NPs were uniformly dispersed on the pore wall of composite hydrogels, and incorporated NPs were increased with increasing HAP concentration. Therefore, the distribution of pores was uniform and this morphology resembles that of previously studied pore structures obtained by radiation technique [17]. The pore size of various hydrogels ranged between 130 and 250 μm (average pore size 161 ± 42 μm). To corroborate the presence of HAP NPs in SF/HAP composite hydrogels, EDX mapping equipment was used. Figure 3 shows the results of EDX mapping for the hydrogels. The green marked points in the images represent the site of detected Ca elements in HAP NPs. As shown in Fig. 3, Ca elements were not observed in SF-0 (Fig. 3a), but Ca element (green intensity) was well dispersed, and was increased with increasing incorporated HAP NPs contents (Fig. 3b-d). These findings indicate that HAP NPs were appropriately incorporated and well dispersed into the composite hydrogels. In order to further confirm the presence of HAP NPs, SF/HAP composite hydrogels (SF-0, SF-1, SF-2, and SF-3) were characterized by XRD. The XRD spectrum of SF/HAP composite hydrogels showed amorphous silk I conformation. The specific HAP NPs peaks also appeared in all composite hydrogels. The results show that all SF composite hydrogels were successfully generated by intermolecular chemical crosslinking reaction, instead of secondary structural change of SF. Figure 4 shows the XRD spectrum of SF based composite hydrogels.

### Physical and mechanical properties of SF/HAP composite hydrogels

**Fig. 5:**
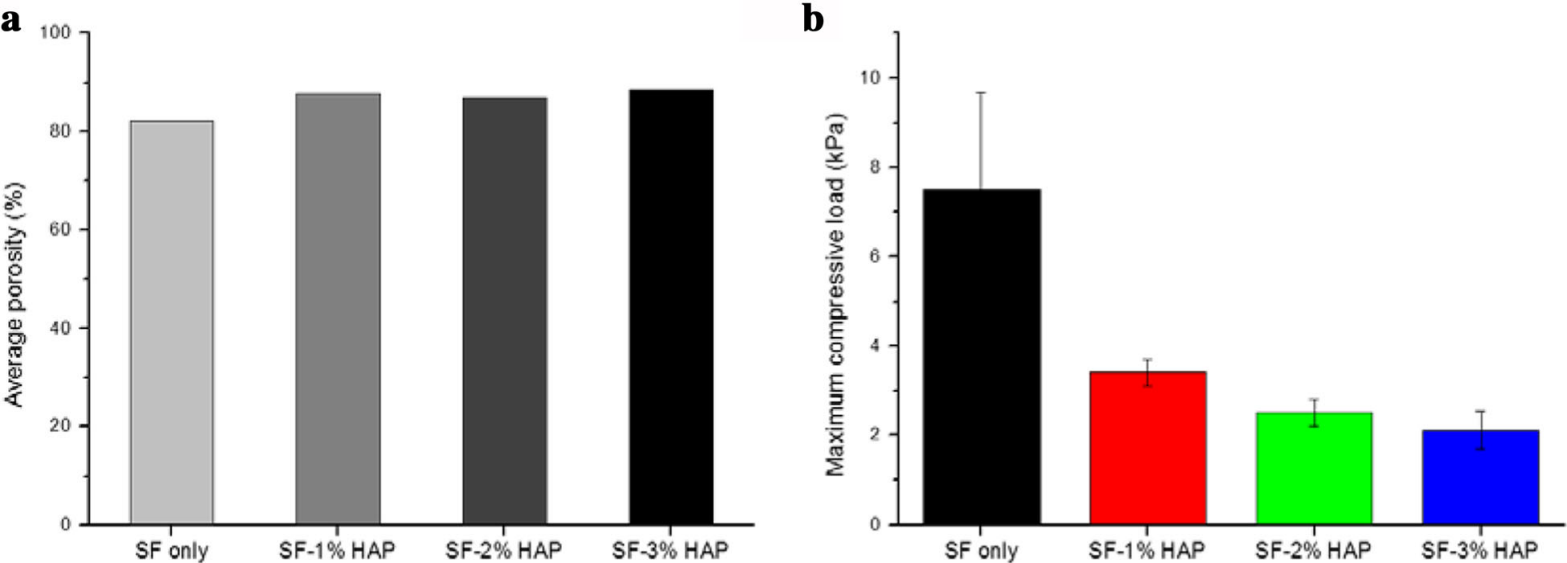
Physical properties of SF/HAP composite scaffolds; **a** porosity and **b** compressive gel strength respectively

Figure 5 describes the porosity and mechanical properties of SF/HAP composite hydrogels. The appropriate pore size and interconnected pores of hydrogels provide sufficient opportunity for cell proliferation. The porosities of SF-0, SF-1, SF-2, and SF-3 were similar (Fig. 5a), and there was no significant difference in the porosity among the hydrogels. Therefore, SF composite hydrogels could provide a good environment for cell migration and differentiation. These results were also related to the pore structure on FE-SEM. Also, Fig. 5b shows the maximum compressive strength of composite hydrogels with/without HAP. Interestingly, SF-0 had the highest compressive strength compared with HAP incorporated SF hydrogels, and also the maximum compressive strength of composite hydrogels decreased as the HAP NPs content increased up to 3 wt% because of the lack of organic/inorganic interaction. Furthermore, during the irradiation, gelation did not occur when more than 3% HAP was added (data not shown). These results were also related to decrease in the compressive strengths of SF/HAP composite scaffolds.

### Cell adhesion and proliferation

**Fig. 6:**
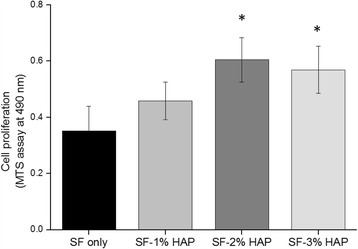
Proliferation of human mesenchymal stem cells on the SF/HAP composite scaffolds evaluated by MTS assay at day 6

**Fig. 7:**
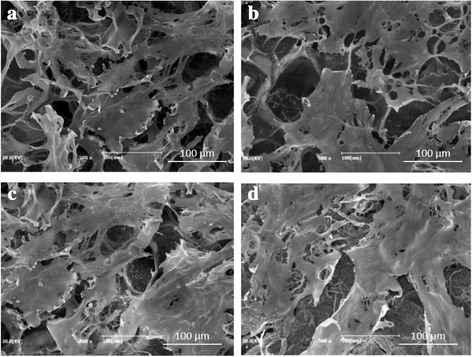
Representative FE-SEM images of hMSCs cultured on **a** SF only, **b** SF-1% HAP, **c** SF-2% HAP, and **d** SF-3% HAP scaffolds at day 6

**Fig. 8:**
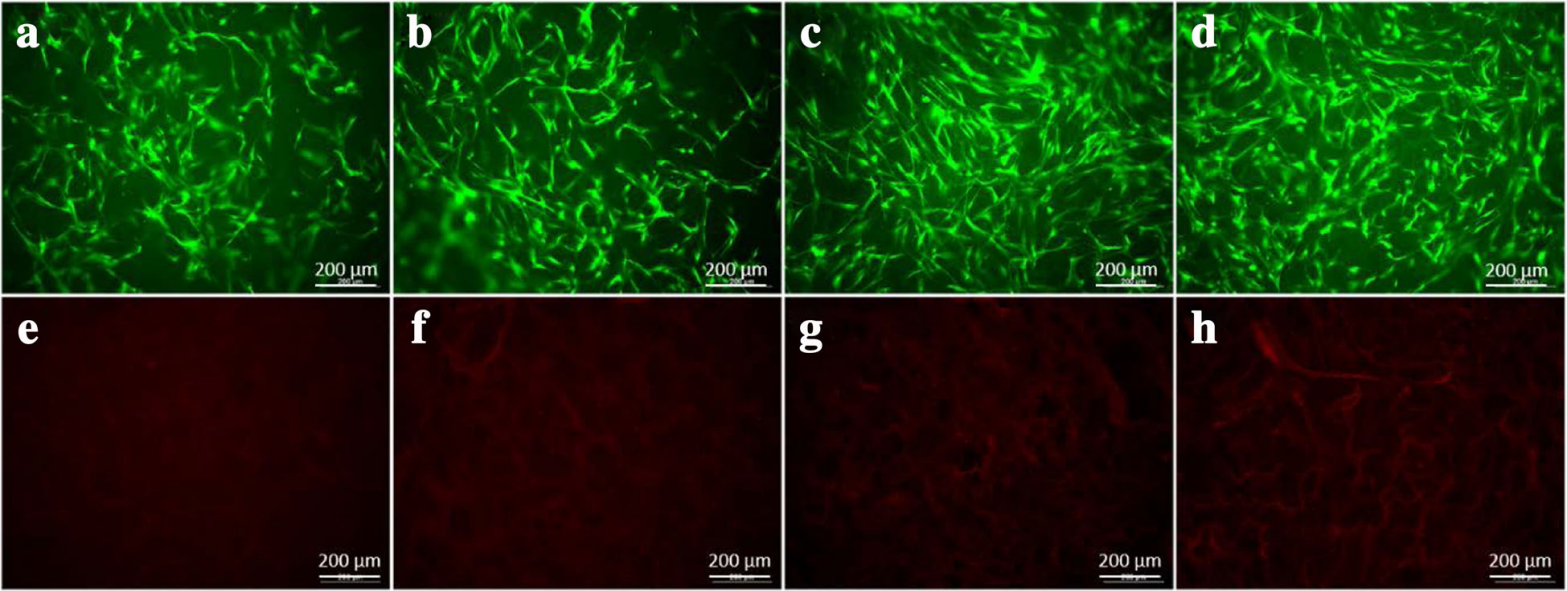
Viability and cytotoxicity staining of cells cultured on **a, e** SF only, **b, f** SF-1% HAP, **c, g** SF-2% HAP, and **d, h** SF-3% HAP scaffolds at day 4

The proliferation and cytotoxicity of the SF/HAP composite hydrogels were determined using the standard MTS assay with hMSCs to evaluate the potential of these materials as a scaffold for bone regeneration. Figure 6 shows that the MTS assay revealed increased cell proliferation rate as the HAP concentration increased, which indicated that HAP supported the proliferation of hMSCs. However, there was no significant difference in proliferation between SF-2 and SF-3. After 6 day of culture, hMSCs were found to have attached and distributed evenly on all hydrogel samples and a small number of hMSCs filled the pores, and formed a continuous monolayer in all hydrogel samples (Fig. 7). The cell monolayer density was increased with increasing HAP NPs concentration. The hMSCs were stained with a Live-Dead™ kit after 4 days of culture, and then observed with confocal microscopy. Green color represents the live cells, while red color represents the dead cells [27]. After 4 days culture, most cells presented green fluorescence, which indicated no significant cell death in the hydrogels under culture, as shown in Fig. 8. The SF/HAP composite hydrogels induced by γ-ray irradiation have noteworthy potential as bone tissue scaffolds, because they showed no significant cytotoxicity against hMSCs.

### Osteogenic differentiation

**Fig. 9:**
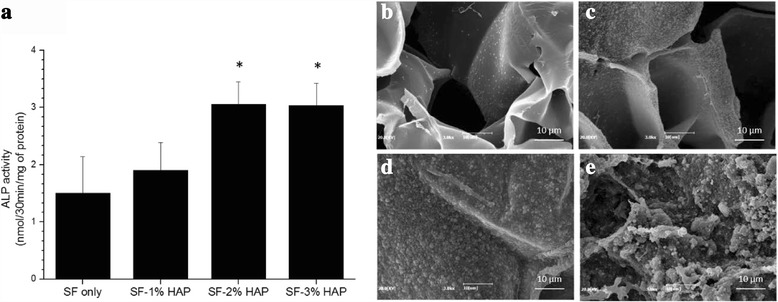
**a** ALP activity of SF/HA hybrid scaffolds and hydroxyl apatite nucleation of **b** SF only, **c** SF-1% HAP, **d** SF-2% HAP, and **e** SF-3% HAP scaffolds in SBF solution at day 7

**Fig. 10:**
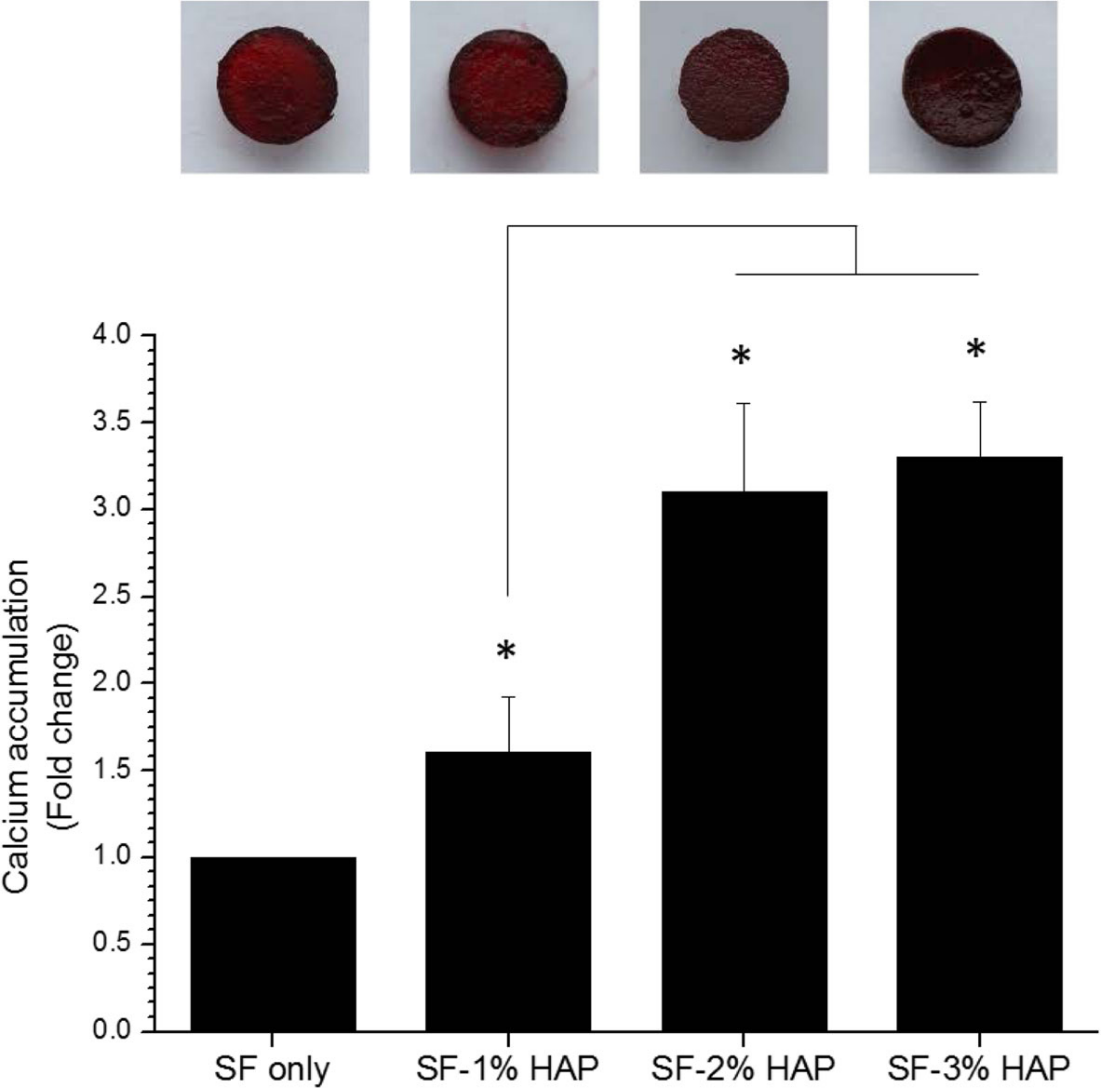
Calcium accumulation of SF/HAP composite scaffolds at day 21

To investigate the osteogenic differentiation of hMSCs seeded on composite hydrogels, ALP activity was assessed. The ALP activity of hMSCs cultured on different types of hydrogel was assessed at 7 days. The ALP activity has been implicated as an early marker of osteogenic differentiation [28–30]. As shown in Fig. 9a, the ALP activity increased as the HAP NPs concentration increased up to 2%. However, there was no significant difference between 2 and 3% HAP concentration. It is considered that the HAP NPs affected osteogenesis and osteogenic differentiation of the hMSCs. Figure 9b-e show SEM imagery of the surface immersed in SBF. After 7 day, the HAP nuclei were formed on the surface of the hydrogels, and then the HAP nuclei grew and the amount of HAP increased with increasing HAP concentration. Figure 10 shows the calcium accumulation of hMSCs-loaded SF/HAP composite hydrogels. The stained Alizarin red-sulfate (AR-S) intensity was increased with increasing HAP concentration. From the results, the SF/HAP composite hydrogels showed excellent cell proliferation, osteogenic differentiation, and calcium accumulation, which are highly desirable properties for bone tissue engineering scaffolds.

## Conclusion

In this study, the SF/HAP composite hydrogels for bone tissue engineering were prepared by gamma-ray irradiation. The morphology and distribution of HAP NPs in the SF hydrogels were investigated by FE-SEM, EDX and XRD. From the results, the SF/HAP composite hydrogels had highly porous structure, and HAP NPs were evenly dispersed in the SF hydrogel. Compared with pure SF hydrogel, the maximum compressive strength of composite hydrogels was decreased with increasing HAP content due to insufficient organic/inorganic interaction. The SF/HAP composite hydrogels also showed increased cell proliferation and adhesion. Furthermore, these hydrogels enhanced in vitro hMSCs osteogenic differentiation. Therefore, these results indicate that the 3D porous SF/HAP composite hydrogel offers promise as a biomaterial for bone tissue engineering.

## Abbreviations

ALP: Alkaline phosphatase activity
AR-S: Alizarin Red-sulfate
*B. mori*: *Bombyx mori*
FBS: Fetal bovine serum
γ-ray: Gamma-ray
HAP: Hydroxyapatite
hMSCs: Human mesenchymal stem cells
NPs: Nanoparticles
PEG: Polyethylene glycol
PVP: Polyvinyl pyrrolidone
SBF: Simulated body fluid
SF: Silk fibroin

## References

[1] Christopher JL, Heather AS, James G, Thomas WS, Janet EH, Barbara EK, Gary SS, Jane BL, Stephen NJ. Osteoblast differentiation and skeletal development are regulated by Mdm2-p53 signaling. J Cell Biology. 2006;172:909–21. 10.1083/jcb.200508130 PMC2063734 16533949

[2] Bhumiratana S, Grayson WL, Castaneda A, Rockwood DN, Gil ES, Kaplan DL, Vunjak-Novakovic G (2011). Nucleation and growth of mineralized bone matrix on silk-hydroxyapatite composite scaffolds. Biomaterials.

[3] Amini AR, Laurencin CT, Nukavarapu SP (2012). Bone tissue eigineering: recent advances and challenges. Crit Rev Biomed Eng.

[4] Bose S, Roy M, Bandyopadhyay A (2012). Recent advances in bone tissue eigineering scaffolds. Trends Biotechnol.

[5] Kim HW, Lee EJ, Jun IK, Kim HE, Knowles JC (2005). Degradation and drug release of phosphate glass/polycaprolactone biological composites for hard-tissue regeneration. J Biomed Mater Res B.

[6] Dhandayuthapani B, Yoshida Y, Maekawa T, Kumar DS. Polymeric scaffolds in tissue engineering application: a review. Int J Polym Sci. 2011:1–19.

[7] Puppi D, Chiellini F, Piras AM, Chiellini E (2010). Polymeric materials for bone and cartilage repair. Prog Polym Sci.

[8] Salgado AJ, Coutinho OP, Reis RL (2004). Bone tissue engineering: state of the art and future trends. Macromol Biosci.

[9] Wang Y, Blasioli DJ, Kim H-J, Kim HS, Kaplan DL (2006). Cartilage tissue engineering with silk scaffolds and human articular chondrocytes. Biomaterials.

[10] Silva SS, Motta A, Rodrigues MT, Pinheiro AFM, Gomes ME, Mano JF, Reis RL, Migliaresi C (2008). Novel genipin-cross-linked chitosan/silk fibroin sponges for cartilage engineering strategies. Biomacromolecules.

[11] Bhardwaj N, Singh YP, Devi D, Kandimalla R, Kotoky J, Mandal BB (2016). Potential of silk fibroin/chondrocyte constructs of muga silkworm Antheraea assamensis for catilage tissue engineering. J Mater Chem B.

[12] Lovett M, Cannizzaro C, Daheron L, Messmer B, Vunjak-Novakovic G, Kaplan DL (2007). Silk fibroin microtubes for blood vessel engineering. Biomaterials.

[13] Zhang X, Baughman CB, Kaplan DL (2008). In vitro evaluation of electrospun silk fibroin scaffolds for vascular cell growth. Biomaterials.

[14] Correia C, Bhumiratana S, Yan L-P, Oliveira AL, Gimble JM, Rockwood D, Kaplan DL, Sousa RA, Reis RL (2012). Development of silk-based scaffolds for tissue engineering of bone from human adipose-derived stem cells. Acta Biomater.

[15] Xiao W, Liu W, Sun J, Dan X, Wei D, Fan H (2012). Ultrasonication and genipin cross-linking to prepare novel silk fibroin–gelatin composite hydrogel. J Bioact Compat Polym.

[16] Huang L, Li C, Yuan W, Shi G (2013). Strong composite films with layered structures prepared by casting silk fibroin–graphene oxide hydrogels. Nano.

[17] Kim MH, Park WH (2016). Chemically cross-linked silk fibroin hydrogel with enhanced elastic properties, biodegradability, and biocompatibility. Int J Nanomedicine.

[18] Im DS, Kim MH, Yoon YI, Park WH (2016). Gelation behaviors and mechanism of silk fibroin according to the addition of nitrate salts. Int J Mol Sci.

[19] Wang P, Qi C, Yu Y, Yuan J, Cui L, Tang G, Wang Q, Fan X (2015). Covalent immobilization of catalase onto regenerated silk fibroin via tyrosinase-catalyzed cross-linking. Appl Biochem Biotechnol.

[20] Ahmed EM (2015). Hydrogel: preparation, characterization, and applications: a review. J Adv Res.

[21] Ahmed EM (2007). Hydrogel: preparation, characterization, and applications: a review. J Adv Res.

[22] Swaroop K, Francis S, Somashekarappa HM (2016). Gamma irradiation synthesis of Ag/PVA hydrogels and its antibacterial activity. Mater Today.

[23] Park CH, Jeong L, Cho D, Kwon OH, Park WH (2013). Effect of methylcellulose on the formation and drug release behavior of silk fibroin hydrogel. Carbohydr Polym.

[24] Min B-M, Lee G, Kim SH, Nam YS, Lee TS, Park WH (2004). Electrospinning of silk fibroin nanofibers and its effect on the adhesion and spreading of normal human keratinocytes and fibroblasts in vitro. Biomaterials.

[25] Kaplan DL, Kim U-J, Park J, Jin H-J. Concentrated aqueous silk fibroin solution and use thereof. US Patents; 2009:US7635755 B2.

[26] Kim BS, Park KE, Kim MH, You HK, Lee J, Park WH (2015). Effect of nanofiber content on bone regeneration of silk fibroin/poly (ε-caprolactone) nano/microfibrous composite scaffolds. Int J Nanomedicine.

[27] Wang Y, Bella E, Lee CSD, Migliaresi C, Pelcastre L, Schwartz Z, Boyan BD, Motta A (2010). The synergistic effects of 3-D porous silk fibroin matrix scaffold properties and hydrodynamic environment in cartilage tissue regeneration. Biomaterials.

[28] Li JJ, Gil ES, Hayden RS, Li C, Roohani-Esfahani S-I, Kaplan DL, Zreiqat H (2013). Multiple silk coatings on biphasic calcium phosphate scaffolds: effect on physical and mechanical properties and in vitro osteogenic response of human mesenchymal stem cells. Biomacromolecules.

[29] Cameron AR, Frith JE, Justin J, Cooper-White JJ (2011). The influence of substrate creep on mesenchymal stem cell behaviour and phenotype. Biomaterials.

[30] Kim J-H, Kim D-K, Lee OJ, Ju HW, Lee JM, Moon BM, Park HJ, Kim DW, Lee JH, Park CH (2016). Osteoinductive silk fibroin/titanium dioxide/hydroxyapatite hybrid scaffold for bone tissue engineering. Int J Biol Macromol.

